# The Emerging Role of Phosphodiesterase Inhibitors in Fragile X Syndrome and Autism Spectrum Disorder

**DOI:** 10.3390/ph18101507

**Published:** 2025-10-08

**Authors:** Shilu Deepa Thomas, Hend Abdulaziz Mohammed, Mohammad I. K. Hamad, Murat Oz, Yauhen Statsenko, Bassem Sadek

**Affiliations:** 1 Department of Pharmacology & Therapeutics, College of Medicine and Health Sciences, United Arab Emirates University, Al Ain P.O. Box 15551, United Arab Emirates; 700039712@uaeu.ac.ae (S.D.T.); 201911882@uaeu.ac.ae (H.A.M.); 2 Zayed Bin Sultan Centre for Health Sciences, United Arab Emirates University, Al Ain P.O. Box 15551, United Arab Emirates; 3 Department of Anatomy, College of Medicine and Health Sciences, United Arab Emirates University, Al Ain P.O. Box 15551, United Arab Emirates; m.hamad@uaeu.ac.ae; 4 Department of Pharmacology and Therapeutics, College of Pharmacy, Kuwait University, Safat 13110, Kuwait; murat.oz@hsc.edu.kw; 5 Department of Radiology, College of Medicine and Health Sciences, United Arab Emirates University, Al Ain P.O. Box 15551, United Arab Emirates; e.a.statsenko@uaeu.ac.ae

**Keywords:** cyclic nucleotides, phosphodiesterase, phosphodiesterase inhibitor, cyclic GMP, cyclic AMP, neuroinflammation, clinical trials

## Abstract

Autism spectrum disorder (ASD) and Fragile X syndrome (FXS) are neurodevelopmental disorders marked by deficits in communication and social interaction, often accompanied by anxiety, seizures, and intellectual disability. FXS, the most common monogenic cause of ASD, results from silencing of the *FMR1* gene and consequent loss of FMRP, a regulator of synaptic protein synthesis. Disruptions in cyclic nucleotide (cAMP and cGMP) signaling underlie both ASD and FXS contributing to impaired neurodevelopment, synaptic plasticity, learning, and memory. Notably, reduced cAMP levels have been observed in platelets, lymphoblastoid cell lines and neural cells from FXS patients as well as *Fmr1* KO and *dfmr1 Drosophila* models, linking FMRP deficiency to impaired cAMP regulation. Phosphodiesterase (PDE) inhibitors, which prevent the breakdown of cAMP and cGMP, have emerged as promising therapeutic candidates due to their ability to modulate neuronal signaling. Several PDE isoforms—including PDE2A, PDE4D, and PDE10A—have been implicated in ASD, and FXS, as they regulate pathways involved in synaptic plasticity, cognition, and social behavior. Preclinical and clinical studies show that PDE inhibition modulates neuroplasticity, neurogenesis, and neuroinflammation, thereby ameliorating autism-related behaviors. BPN14770 (a PDE4 inhibitor) has shown promising efficacy in FXS patients while cilostazol, pentoxifylline, resveratrol, and luteolin have showed improvements in children with ASD. However, challenges such as isoform-specific targeting, optimal therapeutic window, and timing of intervention remain. Collectively, these findings highlight PDE inhibition as a novel therapeutic avenue with the potential to restore cognitive and socio-behavioral functions in ASD and FXS, for which effective targeted treatments remain unavailable.

## 1. Introduction

Autism spectrum disorder (ASD) is a set of behavioral and neurodevelopmental disorder featuring social and communication deficits accompanied by increased repetitive and/or restrictive behaviors [1]. ASD is often accompanied by various cognitive, and emotional challenges linked to a high prevalence of co-occurring conditions, including attention-deficit hyperactivity disorder (ADHD), epilepsy, anxiety, depression, intellectual disabilities, speech impairments, gastrointestinal problems, sleep disturbances, and sensory processing issues, which complicate diagnosis and management [2,3]. Globally, autism affects about 1–2% of the population, with diagnoses occurring more often in males than in females [4]. Despite advances in the understanding of ASD, reliable diagnostic biomarkers and effective treatments for its core symptoms are still lacking.

Fragile X syndrome (FXS) is the most common inherited cause of intellectual disability and ASD. FXS results from a CGG trinucleotide repeat expansion in the noncoding region of the fragile X messenger ribonucleoprotein 1 (*FMR1*) gene, leading to transcriptional silencing and loss of the fragile X messenger ribonucleoprotein (FMRP). FMRP regulates the translation of numerous synaptic proteins, thereby modulating synaptic plasticity and neuronal signaling [5,6].

The cyclic adenosine monophosphate (cAMP) and cyclic guanosine monophosphate (cGMP) are key second messengers that mediate intracellular signaling in both neurons and glial cells [7]. The balance of cAMP/cGMP signaling is tightly controlled by cyclase enzymes, which generate them, and phosphodiesterases (PDEs), which break down cAMP and cGMP into their respective nucleoside 5′ monophosphates through hydrolysis. The precise spatiotemporal expression of PDEs is crucial for maintaining appropriate cAMP and cGMP levels in different brain regions, thus ensuring proper neuronal function [7]. Dysregulation of cAMP and GMP signaling has been implicated in FXS and ASD, with evidence of reduced cAMP levels in platelets, lymphoblastoid cells and neural cells derived from patients with FXS and animal models (*dfmr1 Drosophila* and *Fmr1*KO mice) [8].

PDE inhibitors have been explored for disorders such as Alzheimer’s disease, dementia, depression, and schizophrenia [7,9]. Recent studies have increasingly highlighted PDEs as potential therapeutic targets in ASD [10,11]. Pharmacological blockade of PDE2A and PDE4D has been linked to various autism-like behaviors in mouse models [12,13,14]. PDE10A inhibition has also been suggested as a potential therapeutic approach for ASD [15]. More recently, PDE4 inhibitor BPN14770 has gained attention in FXS, where preclinical and clinical findings show that their modulation can improve behavioral performance [4]. This review integrates evidence highlighting the role of cAMP signaling and the dysregulation of multiple PDE isoforms (PDE1, PDE2A, PDE4D, PDE10A, PDE11A) in ASD/FXS behavioral phenotypes. It provides a comprehensive overview of both synthetic and natural PDE inhibitors as emerging therapeutic strategies and emphasizes the underlying mechanistic pathways (including oxidative stress, synaptic plasticity and neuroinflammation mediated through increased cAMP and cGMP signaling) that lead to improved behavioral outcomes in ASD and FXS. It further underscores developmental and sex-specific aspects of PDE regulation.

## 2. Cyclic Nucleotide Signaling and PDE Regulation in the Brain

### 2.1. Cyclic Nucleotide Signaling and the Role of PDE

cAMP and cGMP represent the most common second messengers involved in various intracellular signal transduction processes [16]. cAMP and cGMP are essential for neurodevelopment, synaptic plasticity, and cognitive functions such as learning and memory [17,18]. They are also involved in multiple aspects of neural development and function promoting neurogenesis, supporting neurite outgrowth and synapse formation, and regulating spine density [19,20,21]. The second messengers cAMP and cGMP are generated by adenylyl cyclases (AC) and guanylyl cyclases (GC), respectively. They further activate cAMP- and cGMP-dependent protein kinases (PKA and PKG), along with other effectors regulating numerous physiological processes [22]. The intracellular levels of cAMP and cGMP are regulated by enzymes that break them down into their inactive metabolites, 5′AMP and 5′GMP. These enzymes, known as phosphodiesterases (PDEs), are classified into 11 families (PDE1–11) based on their kinetic characteristics, mechanisms of activation, and inhibitor sensitivity (Table 1). Based on their substrate preferences, PDEs are categorized into three groups: cAMP-specific (PDE4, PDE7, and PDE8), cGMP-specific (PDE5, PDE6, and PDE9), and dual-substrate enzymes capable of hydrolyzing both cAMP and cGMP (PDE1, PDE2, PDE3, PDE10, and PDE11) [23,24].

**Figure 1:**
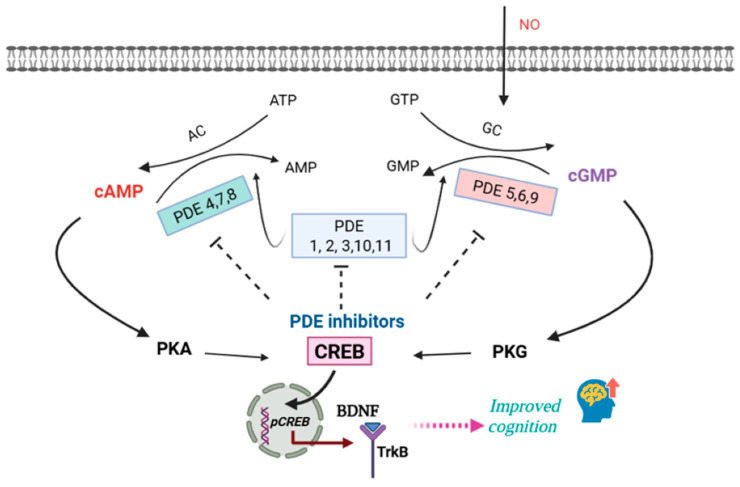
An overview of various PDE subfamilies and cyclic nucleotide signaling.

Both neurons and glial cells use cAMP or cGMP to activate several signaling pathways [25]. The signaling pathway AC/cAMP/PKA regulates neurotransmission by promoting the release of dopamine and glutamate from the presynapse to the postsynapse. In parallel, the GC/cGMP/PKG pathway is activated by nitric oxide (NO). Further, NO diffuses into the dendrites of medium spiny neurons, where it activates soluble guanylyl cyclase (sGC) to produce cGMP [26,27]. Acting as a retrograde messenger, NO enhances the GC/cGMP/PKG cascade and thereby stimulates the synthesis and release of neurotransmitters, particularly glutamate. Phosphorylation of CREB at Ser133 by PKA is a critical step in neuronal plasticity. Once activated, CREB initiates transcription of downstream genes. As a result, proteins such as AMPA receptors and growth factors like BDNF are produced, which are essential for synaptic function and neuroplasticity [28]. An elevation in intracellular cAMP activates PKA, which phosphorylates downstream targets through the Dopamine- and cAMP-Regulated Phospho-Protein MR 32 kDa (DARPP-32) pathway. DARPP-32 acts as a regulator: phosphorylation at Thr34 inhibits protein phosphatase-1 (PP-1), whereas phosphorylation at Thr75 inhibits PKA. This dual regulatory function enables DARPP-32 to either stimulate or inhibit neuroplasticity. Inhibition of PP-1 strengthens PKA-mediated phosphorylation of the AMPA receptor subunit GluR1 and the NMDA receptor subunit NR1, while also regulating numerous downstream effectors such as ion channels (Ca^2+^ and Na^+^), Na^+^, K^+^-ATPase pumps, and transcription factors [29,30]. Figure 1 provides an overview of various PDE subfamilies and cyclic nucleotide signaling.

### 2.2. cAMP Signaling Dysregulation in FXS and ASD

In neurons, cAMP signaling through the transcription factor CREB plays a key role in regulating synaptic transmission, neuronal plasticity, and neurogenesis [31]. Multiple studies suggest disruptions in cAMP signaling in FXS and ASD. The *Drosophila* model of FXS is based on loss of function of *dfmr1*, the *Drosophila* ortholog of *FMR1*. Previous research has demonstrated that cAMP levels are reduced in the brains of *dfmr1* mutant flies and *Fmr1* KO mice [32,33]. Similarly, decreased cAMP levels have been observed in Fragile X patients [34,35]. Remarkably, FMR1 transcription is regulated by CREB-dependent mechanisms in both flies and mammals, which is driven by the cAMP pathway [32,36]. Consistent with these findings, a positive correlation was seen between FMRP levels and cAMP concentrations in patient-derived cell lines and platelets [8].

These findings have led to the hypothesis that restoring cAMP to normal physiological levels can ameliorate cognitive impairments associated with FXS. Although this approach would not reinstate FMRP expression (which is silenced in patients), it may enhance downstream effectors of the cAMP signaling cascade, which are implicated in learning and memory processes [37]. Building on this evidence, cAMP has emerged as a therapeutic target in FXS. In a study by Choi et al. (2015), Rolipram—a PDE-4 inhibitor that blocks cAMP degradation—was shown to elevate cAMP levels and successfully rescue phenotypic deficits in both mouse and fly models of Fragile X [33].

## 3. Abnormalities in PDE Expression and Activity in ASD and FXS

In the context of ASD and FXS, several PDE genes have been implicated through genetic and behavioral studies. FMRP directly targets the mRNAs of PDE1A, PDE2A, and PDE10A, implicating these enzymes in FXS pathology [38]. Most PDE families are expressed in the nervous system, where they play key roles in neurodevelopment, neuronal excitability, synaptic transmission, and neuroplasticity. Each brain region expresses multiple PDEs; no two enzymes share the same regional distribution [25].

The PDE1 family consists of three genes: PDE1A, PDE1B, and PDE1C. PDE1B is highly expressed in the striatum and the dentate gyrus of the hippocampus. Studies of molecular pathways and neural circuits in ASD demonstrate many mutations occurring in genes highly expressed in the striatum. Striatal enlargement, often associated with repetitive behaviors and motor deficits, is commonly reported in autistic individuals [39,40]. Inherited missense variants in *PDE1B* have also been identified in individuals with ASD [41]. *Pde1b* KO mice display behavioral phenotypes such as hyperactivity and learning deficits, supporting a role for PDE1B in cognitive functions [42,43]. Vinpocetine, a PDE1 inhibitor, has been found to ameliorate ASD-like behaviors in preclinical studies [44].

PDE2A is a dual-substrate enzyme that degrades both cAMP and cGMP and is activated by cGMP. It is found in both the peripheral tissues and the CNS, with notably high levels in the brain [45]. It is particularly concentrated in regions such as the hippocampus, cortex, and striatum, indicating its role in regulating cAMP and cGMP signaling within brain areas crucial for emotion, perception, learning, and memory, functions commonly impaired in individuals with FXS or ASD [46]. Inhibition of PDE2A is known to have pro-cognitive effects [47]. A homozygous splicing mutation in *PDE2A* has been identified in patients with atypical Rett syndrome [48]. PDE2A has also been implicated in the pathophysiology of FXS [49]. PDE2A translation is negatively modulated by FMRP [13]. In the absence of FMRP, as seen in *Fmr1*KO mice, PDE2A expression is elevated, leading to reduced levels of cAMP and cGMP. This disruption in second messenger signaling is associated with exaggerated mGluR-dependent long-term depression (LTD), immature dendritic spine morphology, and deficits in social behavior—the hallmarks of FXS.

Inhibition of PDE2A with BAY60-7550 normalized the heightened mGluR-dependent LTD in the hippocampus of *Fmr1*KO mice. Further, inhibiting PDE2A restored axonal length and immature spine morphology in cultured cortical *Fmr1*KO neurons. Adolescent *Fmr1*KO mice also exhibited improved social interaction after PDE2A inhibition. Notably, early intervention with BAY60-7550 (from postnatal day 5 to 21) in *Fmr1*KO mice resulted in sustained behavioral improvements even at postnatal day 30, following a 9-day drug washout (Maurin et al., 2019). These findings suggest a window for early therapeutic targeting of PDE2A that may lead to long-lasting developmental benefits [49]. Similar therapeutic benefits of PDE2A inhibition were observed in *Fmr1*−^Δ^ *exon8* rats, another preclinical model of FXS. The pharmacological inhibition of PDE2A with Bay 60-7550 improved social and cognitive behaviors [50]. In a separate study using valproic acid (VPA)-exposed rats, elevated cGMP hydrolysis was observed during synaptogenesis (postnatal day 14), when FMRP expression is at its highest. This suggests that PDE2A plays a critical role in cGMP breakdown during early postnatal development, accounting for approximately 50% of the reduction in cGMP levels during synaptogenesis in the rat brain, further suggesting that targeting PDE2A early in development could help correct the abnormal neurodevelopment observed in FXS [50]. To further probe the developmental role of PDE2A, heterozygous *Pde2a*KO (*Pde2a*^+/−^) mice were studied. *Pde2a* deletion in C57BL/6J mice leads to sex-dependent socio-cognitive deficits, with male *Pde2a*^+/−^ mice displaying persistent social impairments and hyperactivity, while females show milder cognitive deficits emerging in adulthood. These behavioral phenotypes are associated with microglial activation, elevated glutathione levels, and increased GluR1 receptor externalization in the CA1 region, resulting in impaired mGluR-dependent long-term depression [51]. Interestingly, this phenotype is linked to elevated cAMP and cGMP levels, contrasting with the reduced second messenger levels observed in FXS [52]. These findings shed new light on the pathophysiology of FXS. FMRP regulates the balance of both cAMP and cGMP levels through its control of PDE2A expression. These findings highlight a potentially critical role for cGMP levels—or the cGMP/cAMP ratio—in the pathophysiology of autism, although further studies are needed to confirm this hypothesis. This further supports the emerging “cAMP and cGMP theory” of FXS, emphasizing the involvement of both second messengers in the disorder [52].

The PDE4 family, comprising PDE4A–D, has distinct isoforms with a non-redundant roles in the brain [53]. Early evidence supporting phosphodiesterase-4 (PDE4) as a therapeutic target in FXS originated from studies in *Drosophila* models [32,33]. This interest was sparked by findings from Berry-Kravis and colleagues, who reported reduced cAMP levels in FXS patients. In *Drosophila*, there is a single gene equivalent to human *FMR1*, and mutational studies have revealed that PDE4 plays a crucial role in a signaling pathway essential for associative learning, courtship behavior, and brain development [38]. Loss of the *dnc* gene, the sole homolog of the mammalian PDE4 genes, impairs learning and memory by blocking cAMP breakdown, which in turn disrupts the normal spatial and temporal regulation of cAMP signaling [34,54,55,56].

Kanellopoulos et al. [32] used *Drosophila* models with heterozygous mutations to demonstrate that FMRP loss leads to elevated metabotropic glutamate receptor (mGluR) activity and decreased cAMP levels and underlies olfactory associative learning and memory deficits. Their findings also reveal that cAMP positively regulates *Fmr1* transcription through the PKA-CREB pathway. They further reported that treatment with rolipram, a PDE4 inhibitor, rescued the learning deficit. Choi and colleagues extended these findings using *Dfmr1* null flies, showing that both rolipram and another PDE4 inhibitor, RO201724, reversed behavioral deficits. cAMP levels can be regulated by mGluR signaling. In both *Drosophila* and mouse models of FXS, they showed that inhibiting PDE4 ameliorates memory impairments and corrects structural defects in the brain. In the mouse model, both acute and chronic PDE-4 inhibition rescued the exaggerated mGluR-dependent long-term depression phenotype in hippocampal slices. Together, the studies by Kanellopoulos et al. and Choi et al. highlight the link between mGluR hyperactivity and cAMP signaling deficits in FXS and validate PDE4 inhibition as a promising therapeutic approach to ameliorate cognitive and synaptic dysfunction in individuals with FXS [32,33].

BPN14770, a PDE4D-negative allosteric modulator, showed therapeutic potential in a mouse model of FXS by improving behavior and dendritic spine morphology. Notably, its benefits persisted two weeks post-treatment, suggesting lasting effects. The role of PDE4D in brain function is further highlighted by rare autosomal dominant mutations that cause a condition called acrodysostosis without hormone resistance (ACRDYS2) [57,58]. This neurodevelopmental syndrome includes features such as short stature, abnormal facial features, intellectual disability, and delayed speech and motor development. Disruption of PDE4 subtypes may underlie key autism-associated comorbidities. *Pde4b*KO mice show reduced prepulse inhibition, heightened amphetamine-induced hyperactivity, impaired associative learning, and increased anxiety-like behavior. These features align with ASD comorbidities such as hyperactivity, learning difficulties, and anxiety disorders. Similarly, *Pde4d* KO mice demonstrate reduced immobility in behavioral despair models, which may relate to depression and affective symptoms occasionally co-occurring in ASD [59,60]. These findings support the idea that PDE4B and PDE4D play roles in molecular pathways contributing to ASD-related neuropsychiatric symptoms, and that subtype selective PDE4 modulation might offer a targeted strategy for managing these comorbidities.

*CC2D1A* is an ASD/ID risk gene that plays an essential role in neuronal development, differentiation, and plasticity. It regulates several signaling pathways, including PDK1/AKT, EGFR/ERK, and CREB. By repressing PDE4D, *CC2D1A* enhances cAMP levels and promotes CREB activation, a process critical for learning and memory. In *Cc2d1a*KO mice, PDE4D hyperactivity leads to excessive cAMP degradation, impaired CREB signaling, and resulting deficits in memory and social behavior [61,62]. Neurodevelopmental disorders show a strong male bias, with prevalence ratios of 4:1 in ASD. In a study on mice lacking the signaling scaffold CC2D1A, researchers uncovered sex-specific molecular and behavioral effects. In male *Cc2d1a*-deficient mice, PDE4D was hyperactive, reducing CREB signaling and impairing spatial memory. These deficits were absent in females. Importantly, inhibiting PDE4D restored cognitive function in males but had no effect in females. These findings suggest that *CC2D1A* controls cAMP signaling in a male-specific manner, contributing to sex differences in neurodevelopmental disorders [14].

Rett syndrome, a severe neurodevelopmental disorder primarily affecting females, is caused by mutations in the *MECP2* gene. It is characterized by deficits in synaptogenesis and neuronal connectivity. Investigations conducted in *Mecp2*-deficient mice, a well-established model of Rett syndrome, revealed reduced resting cAMP levels. The study demonstrated that rolipram, a selective PDE4 inhibitor, increases cAMP and phosphorylated CREB levels in *MECP2* mutant neurons. Treatment with rolipram significantly elevated pCREB expression and effectively rescued several cellular deficits, including reduced dendritic complexity, shortened neurite length, mitochondrial fragmentation, and impaired mitochondrial membrane potential in various *MECP2* mutant neuronal models. This further suggests that excessive cAMP degradation via PDE4 contributes to Rett syndrome pathology, further reinforcing the role of cAMP signaling and PDE regulation in neurodevelopmental disorders, including ASD and FXS [63,64]. Future research should focus on identifying age-specific therapeutic windows to maximize treatment efficacy. Given the observed sex-specific regulation of PDE4D in the brain, future research should account for potential sex-dependent behavioral effects arising from PDE4 modulation.

PDE10A, another dual-specific PDE, plays a critical role in striatal function. Genome-wide association studies have identified a connection between microRNA *miR-137*, an important regulator in human neuropsychiatric conditions and both PDE10A and the development of ASD [65]. *miR-137* is known to be critical for neurogenesis and neuronal development. The heterozygous mice display disrupted synaptic plasticity, along with repetitive behaviors, and deficits in learning and social interaction. Further molecular analyses showed elevated PDE10A levels, a known target of *miR-137*, in these mice. Importantly, behavioral and cognitive impairments were partially reversed by either pharmacological inhibition of PDE10A using papaverine or by genetic knockdown of PDE10A. Papaverine has been found to attenuate neurobehavioral abnormalities in a rodent model of ASD [15]. *Pde10a* KO mice showed a decrease in exploratory locomotor activity and a delay in the acquisition of conditioned avoidance responses [66]. Additionally, it also exhibited a marked alteration in dopamine turnover within the striatum, along with an increased locomotor response to amphetamine. *Pde10a* mRNA is another target of FMRP, as its expression is increased in the brains of *Fmr1*KO mice [13].

PDE11A, which also degrades both cAMP and cGMP, has a splice variant, PDE11A4, that is predominantly expressed in the ventral hippocampus [67,68]. In a study conducted by Hegde et al., 2016, [69] PDE11A was found to be essential for maintaining normal social interactions. Additionally, RNA sequencing pathway analyses highlighted its key regulatory role in the oxytocin signaling pathway and membrane signaling, reinforcing its critical function in social behavior regulation. Table 2 highlights the various PDE isoforms and functional roles in preclinical models of ASD and FXS.

## 4. PDE Inhibitors for Treatment of FXS and ASD

PDE inhibitors have been evaluated for their therapeutic potential in ASD and FXS through studies in animal models and clinical trials. The following section reviews their applications in these neurodevelopmental disorders. Several PDE inhibitors have shown therapeutic potential in ASD and FXS. BPN14770 has progressed through Phase 2 in clinical trials, improving cognition and social behavior in FXS patients. STP1, a novel combination of a PDE inhibitor (ibudilast) and an NKCC1 inhibitor (bumetanide) is currently under evaluation in a Phase 1b clinical trial. Cilostazol used as an adjunct to risperidone in a double-blind, randomized trial was found to be safe and well-tolerated, and significantly improved hyperactivity symptoms. Similarly, pentoxifylline and propentofylline, when co-administered with risperidone in randomized, placebo-controlled trials, produced improvements in behavioral symptoms. Preliminary open-label studies have reported that a liposomal formulation containing the flavonoid luteolin (combined with quercetin/rutin) improved sociability and other behavioral measures in children with ASD (ClinicalTrials.gov: NCT01847521). In addition, resveratrol, a natural PDE inhibitor with anti-inflammatory and antioxidant properties, has shown clinical benefits in small-scale ASD trials. In contrast BAY60-7550, vinpocetine, TAK-063 and other natural PDE inhibitors remain in preclinical studies, showing improvements in social and cognitive phenotypes.

### 4.1. BPN14770 (Zatolmilast)

**Figure 2:**
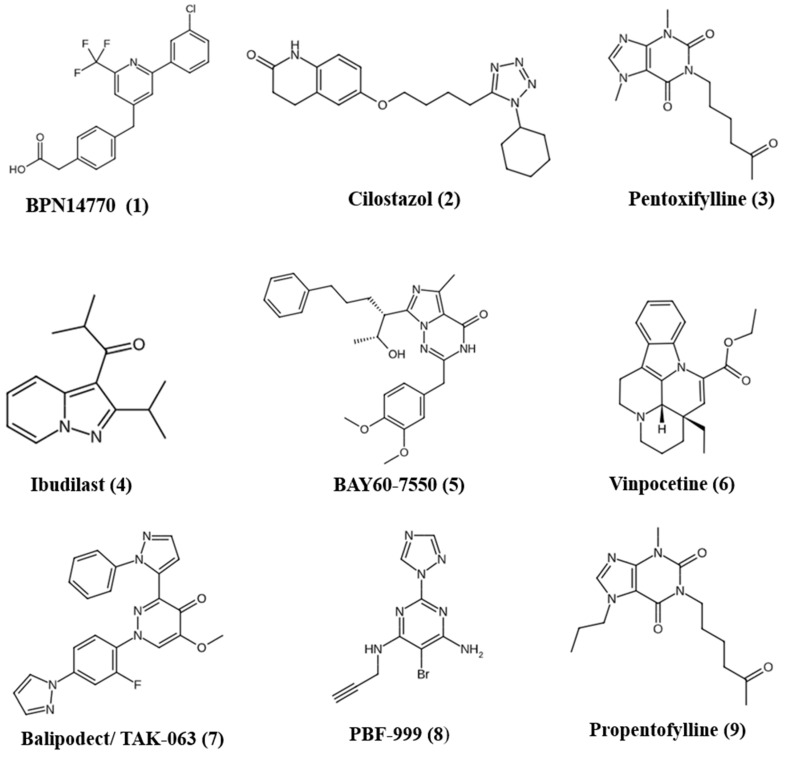

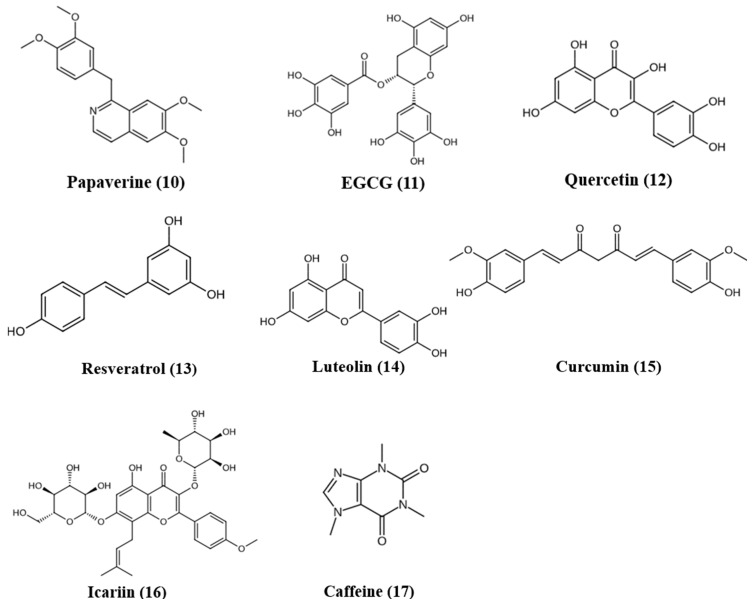
The structures of PDE inhibitors investigated for the treatment of ASD and FXS.

While rolipram, which has been used to treat FXS animal models, inhibits all PDE4 subtypes, BPN14770 (Figure 2(1)) specifically targets the PDE4D subtype. PDE4D, an FMRP target, has shown promise in preclinical and clinical studies. The selective PDE4D inhibitor BPN14770, when administered with a two-week sub-chronic treatment, improves social behavior, naturalistic behaviors (e.g., nesting, marble burying), and dendritic spine morphology in *Fmr1*KO mice [12]. Another study evaluated the effects of chronic BPN14770 in male *Fmr1*KO and wild-type mice. Starting at postnatal day 21, mice received dietary BPN14770 and were tested at day 90 for various behaviors and cerebral protein synthesis. BPN14770 improved several behavioral deficits in *Fmr1*KO mice, including hyperactivity, sleep duration, and social behavior. The treatment also increased regional cerebral protein synthesis in wild-type mice but had limited effects in *Fmr1*KO mice, suggesting genotype-specific regulation via cAMP-dependent pathways [6]. BPN14770 has been evaluated in clinical trials for AD, where it was demonstrated to improve working memory in healthy older adults. However, its development has primarily shifted toward treating FXS [70]. Encouragingly, a Phase 2 clinical trial in adult males with FXS reported positive outcomes for this drug. In a placebo-controlled Phase 2 trial, the drug led to notable cognitive gains in language abilities, along with improvements in caregiver assessments of language and daily functioning [71]. Six weeks after the second treatment phase ended, researchers evaluated the drug’s effectiveness and potential biomarkers by conducting electroencephalographic assessments to measure brain activity. Individuals with FXS typically showed increased N1 event-related potential (ERP) responses to sound, reflecting cortical hyperexcitability. The analysis of trial data showed that decreases in N1 amplitude were linked to higher levels of BPN14770 in the bloodstream, suggesting that the drug may help reduce neural hyperexcitability. Since the N1 ERP is associated with language and behavioral responsiveness, this reduction could explain the observed clinical improvements [72]. Currently, a Phase 2/3 trial in male adolescents (NCT05163808), a Phase 3 trial in adult males (NCT05358886), and an open-label Phase 3 extension study to evaluate long-term safety and tolerability are planned but not yet recruiting. Additionally, early-phase trials are underway for Jordan’s syndrome PPP2R5D (Clinical trial.gov ID: *NCT06717438*). PPP2 syndrome type R5D, also known as Jordan’s syndrome, is a neurodevelopmental condition resulting from pathogenic missense mutations in the *PPP2R5D* gene, which encodes a β-subunit of Protein Phosphatase 2A (PP2A). This disorder is marked by delayed overall development, seizures, macrocephaly, vision problems, low muscle tone (hypotonia), attention deficits, sensory and social difficulties often linked to autism, sleep disturbances, and problems with feeding [73].

### 4.2. Cilostazol

Cilostazol (Figure 2(2)), an anticoagulant commonly used for peripheral artery occlusion, stroke prevention, and post-angioplasty care, works by inhibiting PDE3, thereby increasing intracellular cAMP levels and promoting antiplatelet, vasodilatory, and antithrombotic effects. These properties have led to its repurposing for conditions like Raynaud’s phenomenon, Alzheimer’s disease, and COVID-19 [74,75]. Cilostazol enhances the expression of c-fos and insulin-like growth factor 1 and neurogenesis in the hippocampus [76]. In a double-blind, placebo-controlled, randomized trial evaluating its use in children with ASD, 61 children (aged 5–11 years) received either cilostazol (50 mg/day, increased to 100 mg/day) or placebo in addition to risperidone. Cilostazol showed a significant benefit specifically in reducing hyperactivity symptoms, particularly among children with higher baseline hyperactivity. The treatment was well-tolerated, suggesting cilostazol may be a safe and effective adjunct for managing hyperactivity in this subgroup of ASD patients [77].

### 4.3. Pentoxifylline

Pentoxifylline (Figure 2(3)) is a methylxanthine compound with antioxidant and anti-inflammatory effects. Its anti-inflammatory action mainly occurs through the nonselective inhibition of phosphodiesterase enzymes and activation of adenosine A2 receptors, which elevate cAMP levels. The rise in cAMP activates protein kinase A (PKA), which then inhibits NF-κB nuclear translocation, thereby reducing the production of pro-inflammatory cytokines [78]. This promotes the release of 5-HT and inhibits its reuptake, thereby enhancing serotonergic activity in the CNS. Pentoxifylline’s ability to suppress inflammatory responses, especially by inhibiting TNF-α, is believed to help reduce irritability associated with ASD [79]. Both clinical and preclinical studies suggest that pentoxifylline may offer therapeutic benefits in ASD through its anti-inflammatory and neuroprotective effects. In a 10-week randomized, double-blind, placebo-controlled clinical trial, children with autism who received pentoxifylline alongside risperidone showed significantly greater improvement in behavioral symptoms such as irritability, hyperactivity, stereotypy, and social withdrawal compared to those receiving risperidone with placebo [80]. Supporting this, a preclinical study using a propionic acid-induced rat model of autism demonstrated that pentoxifylline treatment reduced autism-like behaviors, decreased TNF-α levels, and increased brain levels of ATP and nerve growth factor. Additionally, histopathological analysis showed reduced oxidative stress and preservation of hippocampal and cerebellar neurons. Together, these findings highlight pentoxifylline’s potential to improve ASD symptoms by modulating neuroinflammation and supporting neuronal health [81].

### 4.4. Ibudilast

Ibudilast (Figure 2(4)) is a broad-acting PDE inhibitor that primarily targets PDE3, PDE4, PDE10, and PDE11 and exhibits anti-inflammatory and neuroprotective effects by modulating glial cell activity and reducing inflammatory cytokines, nitric oxide, and superoxide levels [82,83]. In a preclinical study using a prenatal valproic acid (VPA)-induced rat model of ASD, ibudilast treatment significantly improved social interaction, spatial learning, and memory, and reduced anxiety, hyperactivity, and pain sensitivity. It also lowered oxidative stress and pro-inflammatory markers (IL-1β, TNF-α, IL-6), reduced GFAP-positive cells and mitigated neuronal damage, suggesting its potential therapeutic role in ASD [84]. A second preclinical study using computational methods identified a combination therapy of ibudilast and gaboxadol. Gaboxadol is a GABA_A_ receptor agonist with a preference for extrasynaptic GABA_A_ receptors. In a FXS mouse model, ibudilast alone improved cognitive performance, while gaboxadol reduced behaviors such as hyperactivity, aggression, anxiety, and stereotypy [85]. Importantly, the combination therapy rescued both cognitive and behavioral phenotypes more effectively than either drug alone, without tolerance or adverse effects upon chronic use. This dual-target approach—modulating both cyclic AMP and GABAergic pathways—offers a promising strategy to address the complex and heterogeneous symptoms of FXS and idiopathic autism.

Abnormalities in intracellular chloride concentration, regulated by NKCC1 and KCC2, have been implicated in neurological disorders such as Down syndrome and ASD. In ASD, disruption of the NKCC1/KCC2 equilibrium leads to impaired chloride homeostasis, causing GABA, a typically inhibitory neurotransmitter to act in an excitatory manner [86,87,88]. While the NKCC1 inhibitor bumetanide improves ASD-related behaviors in clinical studies, its strong diuretic effects due to NKCC2 inhibition limit long-term use [89,90,91,92]. These side effects raise significant concerns about safety and long-term adherence to treatment. Given the multifactorial nature of ASD, the development of multitarget-directed ligands represents a particularly promising therapeutic strategy. This approach is exemplified by the ongoing evaluation of STP1, a novel combination of a PDE inhibitor (ibudilast) and an NKCC1 inhibitor (bumetanide), in a phase 1b clinical trial assessing safety, tolerability, pharmacokinetics, and EEG-based target engagement in subgroup of patients with ASD [93] (Clinicaltrials.gov: NCT04644003). The trial showed good tolerability and improved neural synchronization in ASD patients.

### 4.5. BAY60-7550

Studies have shown that BAY60-7550 (Figure 2(5)), a PDE2A inhibitor, possesses anxiolytic effects and enhances learning and memory and neuronal plasticity in models of neurodegenerative diseases [94,95]. As mentioned earlier, in *Fmr1*KO mice, PDE2A expression is elevated in the cortex and hippocampus, leading to reduced cAMP and cGMP levels. Both acute and chronic administration of BAY607550 effectively reversed the socio-cognitive impairments, restored immature dendritic spine morphology in cortical neuron, and rescued exaggerated mGluR-dependent long-term depression (LTD) in the hippocampus observed in *Fmr1*KO mice [49]. Investigations by Schiavi et al. [50], explored PDE2A’s role in ASD pathology using two validated rat models—genetic (*Fmr1*^Δ^*exon* 8) and environmental (prenatal exposure to VPA). Both models received treatment with PDE2A inhibitor BAY60-7550 before behavioral testing. Socio-communicative behaviors were evaluated using ultrasonic vocalization and three-chamber social interaction tests, while cognitive deficits were assessed through novel object recognition and inhibitory avoidance tasks. Elevated PDE2A activity was observed in VPA-exposed rats early in development. Notably, inhibiting PDE2A with BAY60-7550 corrected communication, social interaction, and cognitive impairments in both models. These findings underscore PDE2A’s critical role in neurodevelopment and support PDE2A inhibition as a promising therapeutic strategy for overlapping deficits in FXS and ASD. No clinical trials testing Bay 60–7550 have been reported so far for ASD or FXS.

### 4.6. Vinpocetine

Vinpocetine (Figure 2(6)), a clinically used drug for preventing cerebrovascular diseases and dementia, has demonstrated strong neuroprotective effects in rodent models [44,96]. Vinpocetine, derived from the periwinkle plant, acts as an inhibitor of PDE1 [97]. It exerts anti-inflammatory effects by preventing the activation of NF-κβ. Additionally, VPN has anti-platelet properties that help enhance cerebral blood flow and support brain metabolism. A study conducted by Luhach et al. evaluated the effects of vinpocetine on autistic behaviors induced by prenatal VPA exposure. Vinpocetine treatment improved deficits in social interaction, repetitive behavior, anxiety, locomotion, and pain sensitivity. It also restored key markers of neuronal function (BDNF, synapsin-IIa, DCX, pCREB), and reduced inflammation and oxidative stress across various brain regions [44]. While various core symptoms of ADHD have been replicated in rodent models, locomotor hyperactivity is the most extensively examined. Evidence from ADHD models suggests that disruptions in cAMP and cGMP signaling pathways are key contributors to this hyperactive behavior [98,99]. Vinpocetine was shown to reduce lead-induced hyperactivity in female mice. While lead-exposed males showed no change in activity, females exhibited significant hyperactivity, which was effectively reversed by vinpocetine. These results highlight vinpocetine’s potential to counteract hyperactive behavior through modulation of cyclic nucleotide signaling pathways [100].

### 4.7. Balipodect/TAK-063

Balipodect/TAK-063 (Figure 2(7)) is a very potent PDE10A inhibitor. TAK-063 exhibited antipsychotic-like properties in rodent models treated with methamphetamine or MK-801 [101]. Additionally, it enhanced performance across various cognitive areas, including recognition memory, attention, working memory, and executive functioning. Using *Fmr1* KO mice, researchers tested TAK-063, to assess its effects on electroencephalographic (EEG) biomarkers. The findings suggest that TAK-063 effectively enhances auditory processing and normalizes EEG biomarkers in *Fmr1* KO mice, supporting its potential for further translational research in the treatment of FXS [102].

### 4.8. PBF-999

PBF-999 (Figure 2(8)) is a dual antagonist of adenosine receptor A2A and PDE10 [30]. Clinical trials are underway to assess the safety and efficacy of PBF-999 in people with Prader–Willi Syndrome, a rare genetic neurodevelopmental disorder that results from the absence of gene expression of the paternally inherited genes on the 15q11.2-q13 chromosome (clinicaltrials.eu ID: 2022-501462-22-00).

### 4.9. Propentofylline

Propentofylline (Figure 2(9)) is a xanthine derivative and a non-selective PDE inhibitor. It also blocks adenosine reuptake and inhibits acetylcholinesterase. In preclinical studies, propentofylline demonstrated the ability to reduce several Alzheimer’s disease-related features, including Aβ plaque accumulation, tau hyperphosphorylation, activation of GSK-3β, microglial production of reactive oxygen species and glutamate, and LPS-induced secretion of pro-inflammatory cytokines like IL-1β and TNF-α. In a randomized, double-blind, placebo-controlled clinical trial involving 48 children with ASD receiving risperidone, propentofylline was assessed as an add-on therapy. Children treated with propentofylline showed greater improvement in Childhood Autism Rating Scale (CARS) scores compared to those in the placebo group [103]. Table 3 and Table 4 summarize the various clinical and preclinical investigations for classical PDE inhibitors that are effective in ASD and FXS.

## 5. Natural Products as PDE Inhibitors

In recent years, natural products have been widely studied for their multi-targeted activities under pathological conditions. These natural products are derived from plants and often function as non-selective PDE inhibitors. Table 5 summarizes the various preclinical investigations for natural PDE inhibitors effective in ASD and FXS.

### 5.1. Papaverine

Papaverine (Figure 2(10)), a PDE10-selective inhibitor, is an opium-derived alkaloid commonly used as an antispasmodic to treat visceral and vascular spasms [106]. Treatment with papaverine elevated intracellular levels of cAMP and NAD^+^, enhanced the expression of CREB, BDNF, and synaptic proteins, and restored mitochondrial membrane potential in neurons exposed to quinolinic acid [107]. It has shown promising neuroprotective and therapeutic effects in models of ASD and ADHD. Papaverine improved core ASD-related behaviors such as social deficits, repetitive behavior, anxiety, hyperactivity, and reduced pain sensitivity. It also restored levels of key neuronal markers linked to neurogenesis (DCX), neuronal survival (BDNF), synaptic function (synapsin-IIa), transcription (pCREB), anti-inflammatory cytokines (IL-10), and antioxidant defense (GSH), while reducing pro-inflammatory markers (IL-6, TNF-α) and oxidative stress across various brain regions. Strong correlations were found between behavioral and biochemical changes, highlighting PDE10A as a potential therapeutic target for ASD [15]. They further examined the effects of papaverine in a rat model of autism induced by developmental hyperserotonemia. Papaverine treatment (15 and 30 mg/kg) brought similar improvements in a hyperserotonemia-induced autism model as well. These results suggest that PDE10A inhibition may help correct ASD-related behavioral and biochemical abnormalities by improving neuronal function, reducing inflammation, and mitigating oxidative stress [108]. Additionally, papaverine was also found to improve ADHD-like behaviors, highlighting PDE10A as a potential therapeutic target for ADHD [109].

### 5.2. (−)-Epigallocatechin-3-gallate (EGCG)

(−)-Epigallocatechin-3-gallate (EGCG) (Figure 2(11)) nonselectively inhibits PDE1-5 [110]. Green tea extract (*Camellia sinensis*) is rich in flavonols such as catechins, epicatechin, epigallocatechin, epicatechin-3-gallate, and derivatives like kaempferol, quercetin, and myricetin. Treatment with green tea extract has been shown to improve behavioral abnormalities and oxidative stress in valproate-induced autism models [111,112]. EGCG has potent antioxidant and anti-inflammatory effects and has been shown to beneficially modulate the gut microbiota in ASD. Autistic individuals often display gut dysbiosis, characterized by increased *Clostridium* and *Prevotella copri* and reduced *Akkermansia muciniphila* and *Bifidobacterium spp.* EGCG counteracts these alterations by inhibiting pathogenic bacteria such as *Clostridium perfringens* and *Clostridium difficile* while enhancing beneficial species like *Bifidobacterium* and *Akkermansia*. This further improves metabolite production, supports intestinal epithelial integrity, and contributes to better brain function, making EGCG a promising complementary strategy for managing ASD-related microbiota imbalances and associated symptoms [113]. EGCG also promoted neurogenesis and plasticity in a Down syndrome mouse model, showing potential in alleviating autistic symptoms [114].

### 5.3. Quercetin

Quercetin (Figure 2(12)) is a naturally derived PDE4-selective inhibitor found in fruits, vegetables, and tea. Quercetin, a potent dietary antioxidant, has been found to alleviate autism-like symptoms by targeting oxidative stress and neuroinflammation. The potential neuroprotective effects of quercetin were observed in a VPA-induced rat model of autism. Prenatal quercetin treatment mitigated both the behavioral and biochemical alterations observed in VPA-exposed rats. Behavioral assessments, including open field and social interaction, were conducted on offspring aged 30–40 days [115]. Brain regions such as the cerebral cortex, hippocampus, striatum, and cerebellum were analyzed for oxidative stress markers. Offspring exposed to VPA showed impaired weight gain, delayed responses in behavioral tests, decreased social interaction, and increased oxidative stress, particularly in the hippocampus and striatum. Prenatal quercetin treatment mitigated these behavioral and biochemical alterations. In another rat model of autism, quercetin treatment significantly reduced brain levels of oxidative stress markers and inflammatory cytokines. It also preserved neuronal and Purkinje cells, which were diminished by propionic acid exposure. Quercetin also improved social interaction and learning abilities in treated rats [116]. These results suggest that quercetin’s neuroprotective effects stem from its ability to reduce oxidative damage and inflammation in the brain. Developmental hypothyroidism disrupts brain development by impairing neuronal migration, hippocampal neurogenesis, and white matter formation, including axonal myelination and oligodendrocyte accumulation. Maternal hypothyroidism has been linked to ASD. Since processes such as neurogenesis, neurite growth, synaptogenesis, and synaptic plasticity are central to ASD pathology, experimentally induced developmental hypothyroidism is considered a suitable model for studying ASD. A diet enriched with α-lipoic acid and α-glycosyl isoquercitrin (AGIQ) mitigated the disrupted neurogenesis by restoring antioxidant enzyme expression, GABAergic and glutamatergic markers, and neuronal populations [117].

### 5.4. Resveratrol

Resveratrol (Figure 2(13)) is a polyphenolic stilbenoid naturally present in grapes, berries, and nuts, known for its anti-neuroinflammatory properties. It weakly inhibited cAMP-specific PDEs, preferably PDE 3 and 4 [118]. It supports neuroprotection partly through its role in enhancing synaptic plasticity [119]. In rat models of autism induced by propanoic acid, resveratrol administered at doses of 5, 10, and 15 mg/kg for four weeks was shown to improve behavioral deficits and reduce brain levels of pro-inflammatory cytokines, including TNF-α and IL-6 [120]. Additionally, prenatal treatment with resveratrol has been reported to prevent social impairments in valproic acid rodent models of autism [121]. A randomized, double-blind, placebo-controlled trial evaluated resveratrol (250 mg twice daily) as an adjunct to risperidone in 62 patients with ASD. The results showed improvements in irritability and related behavioral symptoms, but resveratrol showed no additional benefit for irritability compared to placebo. However, resveratrol was associated with a significant improvement in hyperactivity [104]. Resveratrol is found to exert neuroprotective effects in ASD primarily through antioxidant activity, modulation of mitochondrial function, and anti-inflammatory effects by regulating immune responses [122].

### 5.5. Luteolin

Luteolin (Figure 2(14)) is a non-selective competitive inhibitor of PDE 1-5 [123]. Theoharides et al. reported that dietary supplementation with NeuroProtek^®^, which combines the flavone luteolin with the flavonoids quercetin and rutin, when tested in 37 children with ASD, proved to be safe and well-tolerated, showing beneficial effects by reducing inflammation in both the brain and gut, and enhancing adaptive functioning in children (aged 4–12 y) [105]. In an open-label pilot trial, a combination of luteolin, quercetin, and the quercetin glycoside rutin was reported to significantly improve sociability in children with ASD effectively without significant adverse effects (Clinical Trials. Gov ID: NCT01847521) [124]. Similarly, co-ultramicronized palmitoylethanolamide combined with the flavonoid luteolin effectively alleviated both social and non-social deficits in a murine autism model by modulating TNFα and IL-1β, reducing GFAP and NF-κB expression, and enhancing hippocampal neurogenesis and neuroplasticity [125]. Luteolin exhibits neuroprotective effects through its anti-inflammatory and antioxidant properties, primarily by reducing pro-inflammatory cytokines such as TNFα and IL-1β and inhibiting microglial activation. In LPS-induced neuroinflammatory mouse models, luteolin mitigates neuroinflammation, neuronal damage, and cognitive deficits, highlighting its potential as a therapeutic agent for inflammation-related impairments in autism [126,127].

**Table 5:** A summary of preclinical investigations of natural PDE inhibitors in the management of ASD and FXS.

Preclinical Investigations of Natural PDE Inhibitors Effective in ASD and FXS			
Compound	Dose/Model	Outcomes	References
Papaverine	3/10/30 mg/kg; VPA model.	Improved ASD-related behaviors. Increased BDNF, DCX, pCREB, IL-10, and GSH. Decreased TNF-α, IL-6, and TBARS in brain.	[15]
	15/30 mg/kg; Developmental hyperserotonemia.	Corrected ASD-related behavioral phenotypes, increased BDNF, IL-10, and GSH; decreased TNF-α, IL-6, and TBARS.	[108]
EGCG (green tea catechin)	75 and 300 mg/kg orally; mice VPA model.	Improved behavioral deficits at 300 mg/kg. Reduced oxidative stress.	[111]
Quercetin	50 mg/kg orally; rat VPA model.	Prevented behavioral deficits, mitigated oxidative stress in hippocampus and striatum.	[115]
	80 mg/kg; rat propionic acid model.	Reduced oxidative stress, neuroinflammation (↓TNF-α), preserved Purkinje cells and neuronal populations. Improved social behavior and learning deficits.	[116]
	α-Glycosyl Isoquercitrin (5000 ppm) and α-lipoic acid (1000 ppm in diet); developmental hypothyroidism-induced rat ASD-like model.	Restoration of disrupted hippocampal neurogenesis. AGIQ restored antioxidant enzyme genes.	[117]
Resveratrol	5, 10, 15 mg/kg orally for 28 days; rat propanoic acid model.	Suppressed oxidative/nitrosative stress, mitochondrial dysfunction, TNF-α, and MMP-9. Improved behavioral deficits.	[120]
Co-ultramicronized Palmitoylethanolamide + Luteolin (co-ultraPEA-LUT)	1 mg/kg; mice VPA model.	Improved social and nonsocial behaviors in mice. Neuroprotective and anti-inflammatory effects attributed to mast cell and microglial modulation.	[125]
Curcumin	50, 100, 200 mg/kg, orally for 28 days; rat propanoic acid model.	Suppressed oxidative/nitrosative stress, mitochondrial dysfunction, TNF-α, and MMP-9. Improved social interaction and reduced stereotypy.	[128]
	25, 50, 100 mg/kg; BTBR mice.	Ameliorated social deficits without affecting locomotion or anxiety and restored oxidative stress markers (SOD, CAT) in hippocampus and cerebellum.	[129]
Icariin	80 mg/kg; BTBR mice.	Ameliorated social deficits, repetitive stereotypical behaviors, and short-term memory deficits; reduced hippocampal neuroinflammation. Rescued excitatory-inhibitory synaptic imbalance by reducing vGlut1 without affecting vGAT.	[130]
Caffeine	1 mg/mL; male rat pups prenatally exposed to VPA.	Improved learning and memory, reduced anxiety-like behaviors, and enhanced social interaction deficits.	[131]

↓ denotes decrease.

### 5.6. Curcumin

Curcumin (Figure 2(15)), a polyphenol derived from *Curcuma longa*, exhibits strong anti-inflammatory and anti-cancer properties. In a study by Kuo et al., the therapeutic potential of curcumin was evaluated in *Tsc2* knockout mice [132]. Oral administration of solid lipid curcumin particles reversed recognition memory deficits, highlighting its therapeutic efficacy in TSC. Similarly, in rats with propionic acid (PPA)-induced autism, daily treatment with curcumin at doses of 50, 100, and 200 mg/kg for four weeks improved behavioral performance while alleviating oxidative–nitrosative stress, mitochondrial dysfunction, and the elevation of TNF-α and MMP-9 [128]. In another study in a BTBR model of autism, curcumin ameliorated sociability deficits, with improvements in oxidative stress markers in the hippocampus and cerebellum [129].

### 5.7. Icariin

Icariin (Figure 2(16)) is a cGMP-specific PDE5 inhibitor [133]. Icariin, a major flavonoid found in *Epimedium*, commonly known as Horny Goat Weed, exhibits antioxidant, anti-inflammatory, and anti-apoptotic activities, supporting its potential therapeutic effects in neurological disorders including cerebral ischemia, Alzheimer’s disease, Parkinson’s disease, multiple sclerosis, and depression [134]. Icariin also exerts beneficial effects across cardiovascular and respiratory diseases by modulating NF-κB, MAPK, and PI3K/Akt pathways, reducing cytokine release, lipid accumulation, and immune cell infiltration. In models of atherosclerosis, hypertension, and asthma, it has improved vascular and airway function and attenuated inflammatory damage, highlighting its therapeutic potential [135]. Icariin supplementation (80 mg/kg, 10 days) ameliorated social deficits, reduced repetitive behaviors, and rescued short-term memory deficits in BTBR mice. Icariin reduced hippocampal neuroinflammation by decreasing microglial activation and pro-inflammatory cytokines. It also restored excitatory–inhibitory synaptic balance by lowering elevated vGlut1 levels, further highlighting its potential as a therapeutic agent for ASD [130].

### 5.8. Caffeine

**Figure 3:**
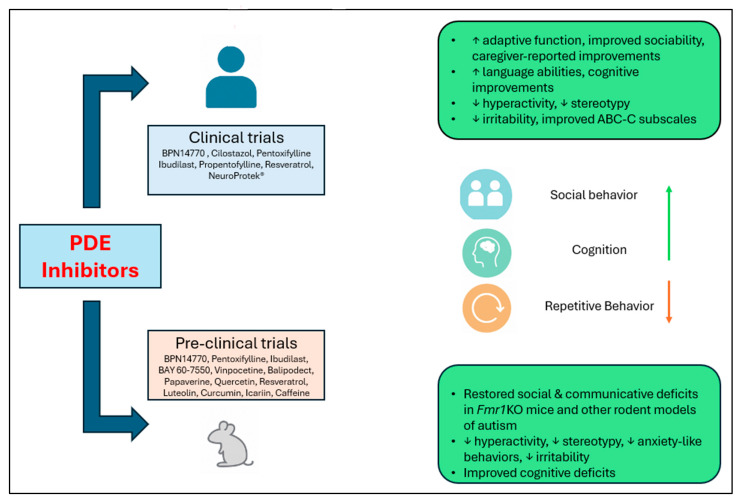
Graphical summary of various behavioral outcomes observed in preclinical and clinical studies of PDE inhibitors as ASD interventions (↑ denotes improvement; ↓ denotes reduction).

Caffeine (Figure 2(17)) is a CNS stimulant and cognitive enhancer, approved by the FDA for treating apnea of prematurity [30]. It acts as a non-selective PDE inhibitor. Investigations have revealed that the cognitive deficits, anxiety-like behaviors, and impaired social interactions induced by prenatal VPA exposure were significantly alleviated by caffeine administration [131]. Figure 3 summarizes the preclinical and clinical outcomes of PDE inhibitors investigated for the treatment of ASD and FXS.

## 6. Possible Mechanisms Underlying the Therapeutic Potential of PDE Inhibition

Despite the diverse causes of ASD, studies consistently highlight disruptions in key cellular processes such as neurogenesis, morphogenesis, synapse formation, and synaptic plasticity. Evidence suggests that impaired neural connectivity and synaptic plasticity may represent a core pathology in both patients and animal models [136]. ASD is also linked to disruptions in neuronal migration and neurogenesis. Changes in neurogenesis can influence synaptic plasticity [137]. Immune dysregulation is a key contributor to ASD, with activated microglia driving inflammation and immune dysfunction. Postmortem analyses of ASD brains reveal altered microglial density and widespread neuroinflammation. Activated microglia and astrocytes in regions such as the hippocampus, cortex, and cerebellum elevate pro-inflammatory cytokines [138,139,140]. Increased levels of pro-inflammatory cytokines have been linked to ASD symptoms in both humans and BTBR mouse models [141,142]. Notably, cerebellar inflammation has been linked to deficits in social interaction and the emergence of repetitive behaviors, highlighting its contribution to autism-related pathology. Patients with ASD show abnormal cytokine expression, including IL-6, TNF-α, IFN-γ. Elevated levels of both innate (IL-1α, TNF-α, GM-CSF, IFNα) and adaptive cytokines (IL-4, IL-13, IL-12p70) are seen especially in children with gastrointestinal symptoms. IL-6 impacts neuronal proliferation, synapse formation, and cognition, and is associated with worsening repetitive behaviors. TNF-α regulates neurogenesis, myelination, and synaptic plasticity, while disruptions in the TNF-α/NF-κB pathway impair neural progenitor proliferation and synaptic development, contributing to ASD features [143,144,145]. Microglia, the brain’s resident immune cells, play a crucial role in maintaining neural health by eliminating pathogens and cellular debris. However, their persistent activation in ASD contributes to sustained neuroinflammation that enhances the expression of NADPH oxidase, more precisely the NOX2 isoform, leading to excessive production of superoxide radicals and increased oxidative damage. The interplay between oxidative stress and microglial activation creates a vicious cycle of neuroinflammation and neuronal damage, which are detrimental to developing brains [146]. Microglia also possess GABA receptors, and disturbances in their expression can disrupt normal cortical developemnt and lead to impaired development of GABAergic neurons, which are key mediators of inhibitory neurotransmisiion, ultimately leading to an imbalance between excitatory and inhibitory signaling, one of the key mechanisms underlying the pathogenesis of neurodevelopmental disorders like ASD [5,147].

PDE inhibition has shown promise in modulating synaptic plasticity in several other neuropsychiatric and cognitive disorders [148,149]. For example, PDE4D inhibition via *miR-139-5p* restored hippocampal neurogenesis and upregulated cAMP/PKA/CREB/BDNF signaling in stress-induced depression models, highlighting its role in enhancing plasticity and antidepressant effects [150]. Studies with PDE4D negative allosteric modulators demonstrated dose-dependent, biphasic enhancement of memory formation and consolidation, accompanied by selective activation of cAMP and synaptic plasticity-related proteins in the hippocampus [151]. *PDE4D*-deficient mice displayed enhanced performance in water maze and object recognition tests, along with increased hippocampal neurogenesis and CREB phosphorylation, effects mimicked by the PDE4 inhibitor rolipram [152]. Long-term potentiation and long-term depression are key electrophysiological mechanisms driving synaptic plasticity through activity-dependent changes in synaptic strength. These processes are regulated by calcium- and GPCR-mediated cAMP/cGMP signaling, which control regulate both transient and prolonged plasticity changes. By modulating these pathways, PDE inhibition can enhance plasticity and support neuronal survival [153].

Deregulation of BDNF levels has been implicated in the pathophysiology of ASD and FXS [154,155]. PDE2 inhibition also improved hippocampal plasticity and BDNF levels [94]. Investigations conducted by Zhao et al. showed that stimulation of gastrointestinal sensory neurons enhanced insulin-like growth factor I (IGF-I) production in the hippocampus, thereby improving cognition in mice. Insulin-like growth factor I (IGF-I) supports cognitive function by promoting angiogenesis and neurogenesis in the hippocampus. The PDE3 inhibitor cilostazol was tested for its ability to boost hippocampal IGF-I, since cAMP is essential for sensory neuron activation. In wild-type mice, cilostazol elevated cAMP and increased hippocampal calcitonin gene-related peptide (CGRP) and IGF-I, and induced c-fos expression in multiple brain regions. Chronic administration also promoted hippocampal angiogenesis and neurogenesis, enhancing spatial learning, further suggesting cilostazol improves cognition by stimulating IGF-I production [76]. Recombinant IGF-I and related compounds have shown therapeutic potential in neurodevelopmental disorders [156]. Fronto-striatal circuit dysfunction contributes to behavioral deficits in FXS, including impaired response inhibition and altered task-related activity in the anterior cingulate cortex and striatum [40]. *Fmr1* KO mice also show synaptic abnormalities in both excitatory and inhibitory transmission in the striatum. Within fronto-striatal circuits, PDE inhibition primarily promotes neuroplasticity and neuroprotection by activating the CREB and DARPP-32 signaling pathways [157]. Collectively, these findings suggest that PDE inhibition can modulate core processes such as neurogenesis, synaptic plasticity, and cognition, which are also impaired in ASD.

PDE inhibitors, by elevating cAMP levels, can exert anti-inflammatory and neuroprotective effects, as low cAMP concentrations favor neuroinflammation through increased cytokine and chemokine release [158]. PDEs influence immune cell activity and the release of pro-inflammatory mediators into the bloodstream. Inhibition of PDE can have beneficial effects by lowering the levels of pro-inflammatory cytokines like IL-1β and TNF-α [159,160]. cAMP modulates immunity by suppressing T-cell activity and promotes neuronal survival via CREB activation. Low cAMP levels trigger NF-κB-mediated pro-inflammatory signaling, driving microglia from a protective M2 to an activated pro-inflammatory (M1) state, which amplifies neuroinflammation [161]. Studies using PDE1 inhibitors have shown promising results, as they suppress NF-κB transcription and activate the cAMP/CREB pathway, thereby reducing neuroinflammation [162]. Inhibition of PDE4 with roflumilast was found to suppress neuroinflammation by enhancing autophagy in microglia. Roflumilast increased LC3-II, reduced p62, and promoted autophagosome formation in BV2 cells, while blocking inflammasome activation, caspase-1 cleavage, and IL-1β production. Importantly, roflumilast reduced neuronal apoptosis via microglial modulation and dose-dependently suppressed IL-1β release. These findings highlight the potential of PDE4 inhibition for suppressing neuroinflammation through autophagy-mediated inhibition of inflammasome activity [163]. Rolipram, a PDE4 inhibitor, has been proven to be effective in various animal models, including depression, neuropathic pain, Alzheimer’s disease, Parkinson’s disease, and multiple sclerosis, for ameliorating neuroinflammation [164]. A study using papaverine, a PDE10 inhibitor, demonstrated significant anti-inflammatory and neuroprotective effects. Papaverine suppressed nitric oxide and cytokine production in LPS-stimulated microglia by elevating cAMP and activating PKA/CREB signaling. These findings highlight PDE10 inhibition as a therapeutic strategy to mitigate neuroinflammation and neuronal damage [165].

**Figure 4:**
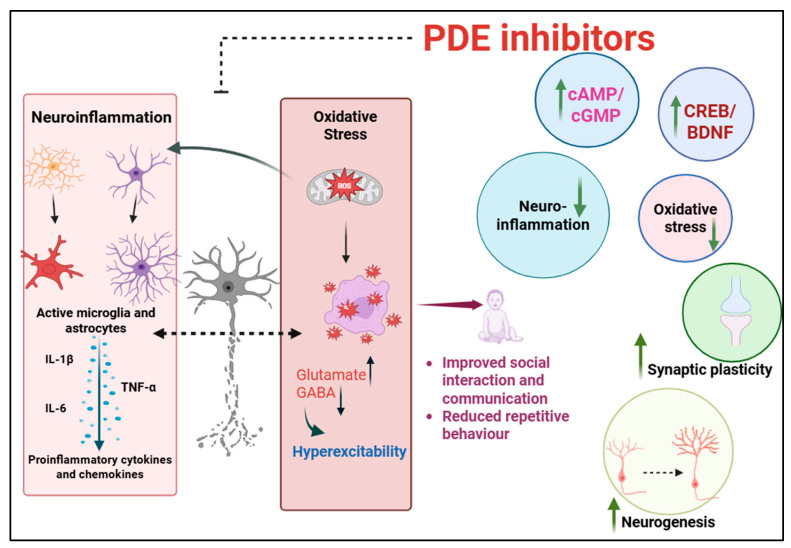
PDE inhibitors restore cAMP and cGMP and enhance CREB/BDNF signaling, which leads to the suppression of neuroinflammation and oxidative stress and enhancement of synaptic plasticity and neurogenesis, thereby improving cognitive and socio-behavioral functions (↑denotes increase; ↓ denotes decrease).

PDE2A, a key regulator of cAMP and cGMP, is dysregulated in FXS and ASD. *Pde2a*^+/−^ mice exhibit sex-dependent socio-cognitive deficits, with males showing severe impairments associated with oxidative stress, microglial activation, and altered glutamate receptor signaling, resulting in disrupted synaptic plasticity [51]. Oxidative stress in *Pde2a*^+/−^ mice reflects an imbalance in redox homeostasis observed in ASD models, where excessive ROS production and impaired antioxidant defense disrupt neuronal signaling and plasticity. Mitochondrial dysfunction, a major source of ROS, may exacerbate oxidative damage and weaken the activity of key antioxidant enzymes such as SOD, CAT, and GPX, promoting microglial activation, lipid peroxidation, and synaptic dysfunction [166,167]. Oxidative and nitrosative stress play a critical role in the development of ASD by disrupting synaptic function. Peroxynitrite and other reactive nitrogen species modify synaptic proteins via nitration and S-nitrosylation, impairing receptor function, vesicle trafficking, and postsynaptic signaling. Long-term potentiation, a key mechanism that underlies learning and memory, is highly vulnerable to oxidative and nitrosative stress. In ASD, impairments in LTP arise from oxidative inactivation of NMDA receptors and nitrosylation of proteins critical for dendritic spine remodeling [168,169]. Elevated levels of reactive oxygen species further disturb the equilibrium between excitatory glutamatergic and inhibitory GABAergic signaling. Oxidative damage to glutamate transporters prolongs synaptic glutamate exposure, thereby enhancing excitotoxic risk. In addition, nitrosative stress diminishes the expression of glutamic acid decarboxylase, the enzyme responsible for converting glutamate to GABA, resulting in decreased GABA synthesis. This disrupts the excitatory–inhibitory balance towards hyperexcitability, as is repeatedly observed in ASD pathophysiology [170]. Dysregulation of GABA receptor function is strongly linked to ASD, with studies reporting altered GABAergic signaling and reduced GABA_A_ receptor activity in autistic brains, contributing to neuronal hyperexcitability and sensory hypersensitivity [171]. Together, these findings suggest that PDE inhibition can modulate key mechanisms underlying ASD pathology, including synaptic plasticity, neurogenesis, and neuroinflammation, supporting the potential of PDE-targeted therapies to restore cognitive and socio-behavioral function. Figure 4 summarizes the key mechanisms through which PDE inhibitors exert therapeutic effects in ASD and FXS.

## 7. Conclusion and Future Perspectives

Neurological and neurodevelopmental disorders, such as Alzheimer’s disease, Parkinson’s disease, FXS, and ASD, remain some of the most challenging diseases in terms of diagnosis, treatment, and long-term management. PDE inhibitors have emerged as having promising therapeutic potential due to their critical roles in intracellular signaling pathways mediated by cAMP and cGMP. In the context of neurodevelopmental disorders, several PDEs—particularly PDE2A, PDE4D, and PDE10A—have been implicated in ASD, FXS, and ADHD. However, the complexity of the PDE family, including isoform-specific effects and regional brain expression, necessitates more precise mapping of their contributions. Moreover, emerging evidence suggests that altered cAMP/cGMP ratios could represent a core pathophysiological mechanism. PDEs do not merely control overall cAMP and cGMP concentrations but organize discrete subcellular nanodomains of cyclic nucleotide signaling, allowing individual cells to respond selectively to diverse inputs. This spatial compartmentalization makes the location and timing of PDE expression as important as its catalytic activity, as both can vary with cell type, developmental stage, or disease status [25]. Therefore, understanding the specific contribution of each PDE isoform to brain development and function requires tools capable of spatiotemporal precision. Though pharmacological studies are essential for translational progress, they are limited to postnatal or adult stages and overlook critical early developmental periods [38]. To develop precise interventions, researchers must identify which PDEs, in which neuronal circuits or compartments, contribute to the pathology. Therefore, future studies using conditional knockout models and optogenetics will be critical in delineating the spatial and temporal roles of individual PDEs during brain development. Conditional knockout models allow for the selective deletion of PDE isoforms in specific neuronal populations. Optogenetics enables real-time modulation of neuronal circuits influenced by PDE signaling and, through subcellular targeting, allows researchers to investigate the function of specific intracellular domains. This will enable the targeted manipulation of PDE expression or activity in specific neuronal circuits and developmental stages, further clarifying how PDE-dependent signaling in distinct neural circuits contributes to neurodevelopmental stages and socio-cognitive behavioral phenotypes. High-precision spatiotemporal control of gene transcription has also been achieved through optogenetics, enabling modulation of endogenous gene expression and epigenetic chromatin modifications [172,173]. The possibility of compensatory adaptive changes among members of the PDE family can also be studied by these methods [174].

Future studies should further investigate the age-dependent effects of PDE modulation, as early-life interventions such as postnatal inhibition of PDE2A have demonstrated long-lasting improvements in behavior. Identifying these critical developmental windows is essential to optimize therapeutic timing and maximize clinical benefits. Additionally, growing evidence of sex-specific regulation of PDE signaling has been observed, particularly in *Cc2d1a*-deficient mice, where PDE4D hyperactivity in males led to impaired CREB signaling and spatial memory deficits, effects that were absent in females. Similarly, *Pde2a*^+/−^ mice exhibited sex-dependent socio-cognitive deficits, with males showing persistent social impairments and hyperactivity, while females displayed milder and delayed cognitive effects. This highlights the importance of considering sex as an important variable in further investigations for ASD and FXS, given their sex-biased incidence, for optimizing PDE-based interventions and advancing personalized medicine approaches.

Since targeting single PDEs in FXS has notable limitations—for instance, PDE4 selectively degrades cAMP without influencing cGMP, and PDE2A shows minimal expression in the cerebellum, a region implicated in FXS—combination therapy can be utilized. PDE4 inhibitors have already demonstrated cognitive and behavioral benefits in Fragile X patients, while PDE2 inhibitors have shown strong preclinical results in animal models, improving social and cognitive function. Moving these findings into human trials is a critical next step. Importantly, combining PDE2 and PDE4 inhibition may provide synergistic effects. Hence, combining multiple isoform-specific drugs into a pathology-tailored therapy may provide additive benefits and help realize the full therapeutic potential of PDE inhibitors. Similarly, it is important to advance our understanding of how to selectively stimulate PDE activity and target the catalytic function of dual-specific PDEs in a functionally selective manner (targeting only their cAMP- or cGMP- hydrolytic activity). By increasing the precision of these interventions, it may be possible to maintain therapeutic efficacy while reducing the side effects that have limited the use of PDE inhibitors.

While most of the current insights into PDE-mediated regulation of neuronal function and behavior and the role of various PDE inhibitors arise from animal models, these may not fully capture the complexity of human neurodevelopmental disorders. These limitations highlight the need for translational validation through clinical studies. Well-planned clinical trials with larger patient cohorts will be essential to confirm the therapeutic potential of PDE modulation, optimize pharmacokinetic properties, and establish optimal age windows, sex-specific responses, and dosing strategies for various neurodevelopmental disorders.

## Figures and Tables

**Table 1:** PDE isoforms and expression pattern in brain.

PDE Family	Isoform	Substrate	Expression in Brain
PDE 1	IA, IB, IC	cAMP and cGMP	1A—striatum, hippocampus
			IB—striatum
			1C—spinal cord, cerebellum
PDE 2	2A	cAMP and cGMP	frontal lobe, hypothalamus, hippocampus, striatum, substantia nigra, amygdala
PDE 3	3A, 3B	cAMP and cGMP	3A—frontal lobe, cingulate cortex, corpus callosum, striatum, cerebellum
			3B—frontal lobe, striatum, cerebellum
PDE 4	4A, 4B, 4C, 4D	cAMP	4A—frontal lobe, parietal lobe, striatum, thalamus, hypothalamus, hippocampus, cerebellum
			4B—frontal lobe, parietal lobe, striatum, thalamus, hippocampus, cerebellum
			4D—frontal lobe, parietal lobe, striatum, thalamus, hippocampus, cerebellum
PDE 5	5A	cGMP	hippocampus, cerebellum
PDE 6	6A, 6B, 6C, 6D, 6H, 6G	cGMP	rod and cone
PDE 7	7A, 7B	cAMP	7A—frontal lobe, temporal lobe, parietal lobe, cingulate cortex, striatum, thalamus, hypothalamus, hippocampus, spinal cord, cerebellum
			7B—striatum
PDE 8	8A, 8B	cAMP	8A—cingulate cortex, corpus callosum, striatum, hypothalamus, spinal cord
			8B—frontal lobe, occipital lobe, striatum, thalamus, hippocampus, pons, medulla oblongata, spinal cord
PDE 9	9A	cGMP	Striatum, hypothalamus, hippocampus, cerebellum
PDE 10	10A	cAMP and cGMP	Striatum, hippocampus, cerebellum
PDE 11	11A	cAMP and cGMP	hippocampus, cerebellum

**Table 2:** Summary of PDE isoforms implicated in ASD and FXS. The findings indicate that dysregulated PDE signaling contributes to impaired cognition, repetitive behaviors, and social deficits. Pharmacological modulation by PDE inhibitors has shown therapeutic potential by restoring synaptic function, improving learning, and ameliorating ASD-related behaviors in animal models.

Isoform	Findings from ASD/FXS Models	Therapeutic Potential of PDE Inhibitor
PDE1B	Inherited missense *PDE1B* variants reported in individuals with ASD; *Pde1b* KO mice show hyperactivity, learning/cognitive deficits	Vinpocetine improves behavioral deficits in rodent models.
PDE2A	Elevated PDE2A in *Fmr1*KO mice reduces cAMP/cGMP, associated with exaggerated mGluR-LTD, immature dendritic spines, and social/cognitive impairments. Heterozygous *Pde2a* KO led to sex-dependent socio-cognitive deficits.	BAY60-7550 normalizes hippocampal mGluR-LTD, restores axonal/spine morphology, and improves social/cognitive behaviors in FXS models; early postnatal treatment produced long-lasting developmental benefits. Timing (developmental window) is critical for efficacy.
PDE4 (B, D)	PDE4 dysregulation contributes to learning/memory deficits. *Drosophila* and mouse FXS models show PDE4 inhibition rescues mGluR-LTD, cognitive deficits, and brain structural defects. *Pde4b* KO mice show hyperactivity, impaired associative learning, anxiety-like behavior; *Pde4d* KO mice show reduced immobility in behavioral despair models. *Cc2d1a* KO mice reveal male-specific PDE4D hyperactivity, reduced CREB signaling, and spatial memory deficits.	Rolipram (PDE4 inhibitor) rescued learning, memory, and synaptic deficits in FXS and Rett syndrome models. BPN14770 (PDE4D negative allosteric modulator) improves behavior and dendritic spine morphology with persistent effects post-treatment. Isoform and sex-specific effects are critical for efficacy.
PDE10A	target of miR-137- critical for neurogenesis and neuronal development. Heterozygous mice show repetitive behaviors, deficits in learning and social behavior, disrupted synaptic plasticity; *Pde10a* KO mice show decreased exploratory activity, delayed conditioned avoidance, altered dopamine turnover. Elevated PDE10A observed in *Fmr1*KO mice.	Papaverine (PDE10A inhibitor) or genetic knockdown partially reverses behavioral and cognitive impairments in rodent models of ASD.
PDE11A	Dual-specific PDE with splice variant PDE11A4 highly expressed in ventral hippocampus. Essential for normal social interactions; RNA-seq analyses show involvement in oxytocin signaling and membrane signaling, highlighting a role in social behavior regulation.	No specific pharmacological inhibitors tested in ASD/FXS preclinical models.

**Table 3:** A summary of clinical investigations on PDE inhibitors in management of FXS and ASD.

Clinical Investigations of PDE Inhibitors in ASD and FXS			
Compound	Population	Outcomes	References/Trial
BPN14770	A 2-period crossover study of BPN14770 in adult males with fragile X syndrome (phase 2).	Cognitive gains in language abilities, along with improvements in caregiver assessments of language and daily functioning.	NCT03569631
	A randomized study of BPN14770 in male adolescents (aged 9 to <18 years) with fragile X syndrome (phase 2/3).	Active, not recruiting.	NCT05163808
	A study of BPN14770 in male adults (aged 18 to 45) with Fragile X syndrome (phase 3).	Completed, no study results posted.	NCT05358886
	An open-label extension study of BPN14770 in subjects with fragile X syndrome (phase 3).	Active, not recruiting.	NCT0536790
Cilostazol	Cilostazol (50–100 mg/day) adjunct to risperidone—61 children (aged 5–11 years) with ASD (double-blind, randomized clinical trial).	Safe and well-tolerated; significant improvement in hyperactivity subscale in children with higher baseline hyperactivity.	[77]
Pentoxifylline	Forty children with ASD (4–12 years); double-blind, placebo-controlled 10-week trial; adjunct to risperidone.	Significant improvement across multiple ABC-C subscales: irritability, social withdrawal, stereotypic behavior and hyperactivity.	[80]
STP1 (Ibudilast + bumetanide)	12 ASD patients (phase 1b, randomized, double-blind, placebo-controlled).	Safe and well-tolerated. EEG markers showed dose-related reduction in gamma power (linked to executive function/memory). Numerical but not statistically significant improvements in clinical scales.	NCT04644003
Propentofylline	Forty-eight children with ASD; randomized, double-blind, placebo-controlled (10 weeks); adjunctive treatment with risperidone.	Adjunctive propentofylline + risperidone showed significant improvement in irritability subscale and CARS scores vs. placebo.	[103]
Resveratrol	Sixty-two patients with ASD; randomized, placebo-controlled (10 weeks); adjunct to risperidone.	Resveratrol significantly improved hyperactivity/non-compliance scores. No significant effect on irritability.	[104]
NeuroProtek^®^ (Luteolin +Quercetin + Rutin)	Thirty-seven children with ASD (4–14 years).	Improved sociability/adaptive functioning; safe and well-tolerated.	[105]
	Fifty children with ASD (4–10 yrs); 26-week open-label trial.	Significant improvement in adaptive functioning; effective in reducing ASD symptoms, with no major adverse effects.	NCT01847521

**Table 4:** A summary of preclinical investigations of classical PDE inhibitors in management of ASD and FXS.

Preclinical Investigations of Classical PDE Inhibitors Effective in FXS and ASD			
Compound	Dose/Model	Outcomes	References
BPN14770	0.3, 1, and 3.0 mg/kg dietary administration; *Fmr1* KO mice	Ameliorated hyperactivity in open field. Improved social behavior.	[6]
	0.3 mg/kg, *Fmr1* KO mice	Improved social interaction, improved dendritic spine morphology. Behavioral improvements persisted 2 weeks after washout.	[12]
Pentoxifylline	300 mg/kg/day, rat PPA model	↓ TNF-α, oxidative stress; NGF; improved autism-like behaviors.	[81]
Ibudilast	5 and 10 mg/kg, VPA rat model	Improved social interaction, learning, memory; ↓ anxiety and hyperactivity, ↓oxidative stress, ↓pro-inflammatory cytokines.	[84]
	Ibudilast (6mg/kg) + Gaboxadol (1.5 mg/kg), *Fmr1* KO mice	Combination treatment rescued both cognitive and behavioral deficits. No adverse effects observed.	[85]
BAY60-7550	0.05 mg/kg at infancy; 0.1 mg/kg adolescence/adulthood *Fmr1*-^Δ^*exon 8* rat, VPA rat model	Improved communicative, social, and cognitive impairments.	[50]
	*Fmr1*KO mouse *Fmr1*KO rat	PDE2A inhibition rescued dendritic spine maturity and exaggerated mGluR-LTD. Restored social and communicative deficits in *Fmr1*KO mice and rats.	[49]
Vinpocetine	10/20 mg/kg, VPA rat model	Improved ASD-like behaviors, increased BDNF, synapsin-IIa, DCX, pCREB/CREB, IL-10, GSH; decreased TNF-α, IL-6, TBARS.	[44]
Balipodect (TAK-063)	0.5 and 5 mg/kg, *Fmr1*KO mice	Improved EEG biomarkers. Normalized cortical auditory processing without depressing baseline EEG power or causing any noticeable sedation or behavioral side effects.	[102]

↓ denotes decrease.

## Data Availability

No new data were created or analyzed in this study. Data sharing is not applicable.
